# Current status of pulmonary artery denervation

**DOI:** 10.3389/fcvm.2022.972256

**Published:** 2022-10-03

**Authors:** Mark G. Davies, Dimitrios Miserlis, Joseph P. Hart

**Affiliations:** ^1^Division of Vascular and Endovascular Surgery, The University of Texas Health at San Antonio, San Antonio, TX, United States; ^2^Division of Vascular and Endovascular Surgery, Medical College of Wisconsin, Milwaukee, WI, United States

**Keywords:** pulmonary denervation, pulmonary hypertension, therapy, outcomes, narrative review

## Abstract

Pulmonary hypertension is a progressive disease with a poor long-term prognosis and high mortality. Pulmonary artery denervation (PADN) is emerging as a potential novel therapy for this condition. The basis of pursuing a sympathetic denervation strategy has its origins in a body of experimental translation work that has demonstrated that denervation can reduce sympathetic nerve activity in various animal models. This reduction in pulmonary sympathetic nerve activity is associated with a reduction in pathological pulmonary hemodynamics in response to mechanical, pharmacological, and toxicologically induced pulmonary hypertension. The most common method of PADN is catheter-directed thermal ablation. Since 2014, there have been 12 reports on the role of PADN in 490 humans with pulmonary hypertension (311:179; treated: control). Of these, six are case series, three are randomized trials, and three are case reports. Ten studies used percutaneous PADN techniques, and two combined PADN with mitral and/or left atrial surgery. PADN treatment has low mortality and morbidity and is associated with an improved 6-minute walking distance, a reduction in both mean pulmonary artery pressure and pulmonary vascular resistance, and an improvement in cardiac output. These improved outcomes were seen over a median follow-up of 12 months (range 2–46 months). A recent meta-analysis of human trials also supports the effectiveness of PADN in carefully selected patients. Based on the current literature, PADN can be effective in select patients with pulmonary hypertension. Additional randomized clinical trials against best medical therapy are required.

## Introduction

Pulmonary hypertension is a progressive condition with a poor prognosis and high mortality in the long term. Pulmonary arterial hypertension is defined as “*mean pulmonary arterial pressure* >*20 mm Hg, an end-expiratory pulmonary artery wedge pressure (PAWP) of? 15 mm Hg, and a pulmonary vascular resistance* >*3 Wood units.at rest”* (1–3). The World Health Organization (WHO) has classified pulmonary arterial hypertension into five clinical subgroups (4, 5) (Table 1).

**Table 1:** Pulmonary hypertension classification.

**Group**	**Etiology**
1	Loss and obstructive remodeling of the pulmonary vascular bed
2	Left-sided heart disease
3	Chronic lung disease
4	Chronic thromboembolic disease
5	Unclear and/or multifactorial mechanisms

Pulmonary arterial hypertension (PAH) (Group 1) is characterized by loss and obstructive remodeling of the pulmonary vascular bed.

Pulmonary hypertension due to left-sided heart disease (LHD) (PH-LHD) (group 2) occurs in response to an increase in left atrial (LA) pressure and is usually a consequence of an underlying cardiac disorder such as HF (with preserved or reduced ejection fraction) or valvular heart disease.

Pulmonary hypertension due to chronic lung disease (CLD) (PH-CLD) and/or hypoxia (group 3) can occur in many lung diseases including chronic obstructive pulmonary disease (COPD), interstitial lung disease, and sleep-disordered breathing.

Chronic thromboembolic PH (CTEPH) (group 4) is characterized by obstruction of the pulmonary vasculature by organized thromboembolic material and vascular remodeling, resulting from prior pulmonary embolism.

Patients with unclear and/or multifactorial mechanisms are listed as group 5.

The mainstays of therapy for pulmonary hypertension are prevention, early identification, and intervention for predisposing etiologies, aggressive medical therapy, and ultimately thoracic transplantation (6, 7). Current approved medical therapies include the use of prostanoids, endothelin-receptor antagonists, soluble guanylate cyclase stimulators, and phosphodiesterase type-5 inhibitors to induce vasodilatation and control pulmonary vascular resistance (8, 9). The current treatment strategy for pulmonary hypertension is based on a multi-parametric risk stratification approach, which incorporates clinical exercise, right ventricular function, and hemodynamic parameters. Patients with pulmonary hypertension are then stratified as low- (<5%), intermediate- (5–10%), or high-risk (>10%) status based on estimated 1-year mortality (10). Given the high mortality and the limited options for therapy, there has been increasing interest in the cellular and molecular processes that lead to pulmonary hypertension. One such set of experiments by Juratsch et al. (11) demonstrated that after surgical dissection of pulmonary artery bifurcation areas, which denervated the area, pulmonary arterial pressure did not increase after pulmonary hypertension was induced by continuous occlusion of the pulmonary artery. These seminal findings have led to an interest in procedures that can induce PADN.

## Anatomy

**Figure 1:**
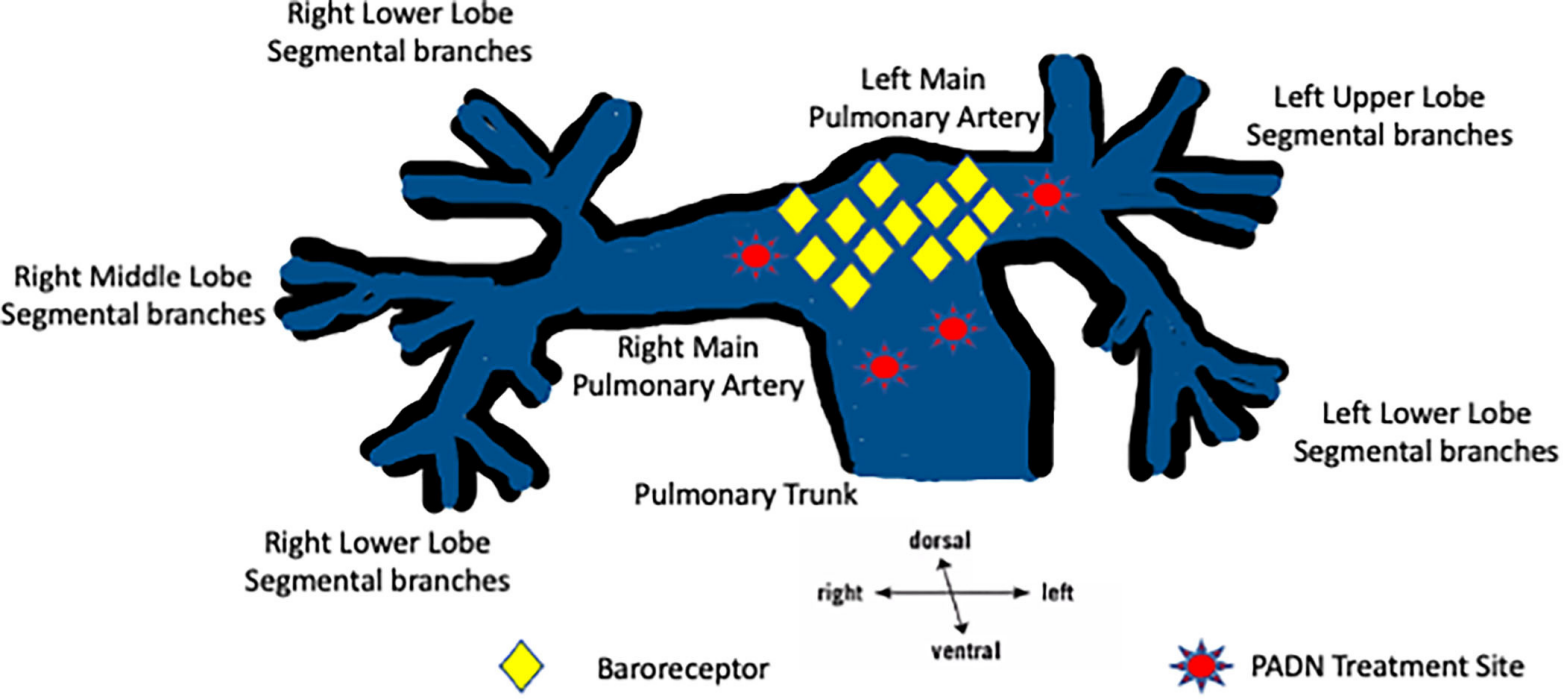
A graphic of the pulmonary arterial tree showing the common locations of baroreceptors and the current PADN treatment sites in the pulmonary trunk, the pulmonary bifurcation, and the pulmonary arteries.

The pattern of innervation of the pulmonary artery follows that of other major vessels in the human body. The coronary plexus of nerves supplies both the sympathetic and parasympathetic nerves to the pulmonary arteries. The sympathetic supply comes from both the cervical and thoracic segments of the spinal cord, while the vagus nerve supplies the parasympathetic innervation. Both the sympathetic and parasympathetic nerve plexi are described as being anterior to the bifurcation of the trachea and posterior to the main pulmonary artery bifurcation. Relevant to this topic on denervation, the pulmonary artery nerve plexus is predominantly derived from the sympathetic system (12). Osorio et al. (13) reported that there are pulmonary baroreceptors at the bifurcation of the pulmonary artery and that the sympathetic nerves supply these baroreceptors. Adrenergic vasomotor fibers have also been reported in the adventitia of pulmonary arteries. Zhang et al. (14) reported, in a canine model, that the majority of the sympathetic nerve plexus could be found in the “*proximal and distal segments of the bilateral pulmonary arteries*” and was predominantly located in the posterior plane of the vessels. A report by Zhou et al. (15), using a canine model, demonstrated that the nerve bundles on the pulmonary arteries originate from several ganglions just above the pulmonary valve on the pulmonary trunk and are found on the left side of the pulmonary arterial trunk. At the pulmonary bifurcation, the nerve trunk divides into left and right branches, which are found on the posterior aspect of the left and right pulmonary arterial branches. Rothman et al. further demonstrated that human pulmonary arteries derive the majority of their innervation from sympathetic nerves (71% vs. 29%; sympathetic vs. parasympathetic) and that >40% of these identified nerve structures are found at a depth of >4 mm from the surface of the luminal side of the pulmonary vessel (16). Figure 1 demonstrates the current understanding of the anatomic location of the baroreceptors and the areas where PADN is performed.

## Pathophysiology

Pulmonary hypertension has been divided into three pathophysiological contexts: precapillary, postcapillary, and combined pre- and postcapillary pulmonary hypertension. Patients with precapillary pulmonary hypertension have increased mean pulmonary arterial pressure (mPAP) but have a normal mean pulmonary wedge pressure (mPWP); this results in a raised transpulmonary gradient (TPG) and increased pulmonary vascular resistance (PVR). An increase in mPWP characterizes patients with postcapillary pulmonary hypertension. They can be distinguished from isolated and combined pre-and postcapillary etiologies based on the PVR and/or diastolic pressure gradient. Pulmonary hypertension is characterized by several biomechanical processes that increase pulmonary vascular resistance: abnormal pulmonary vasoconstrictive activity, small pulmonary arteries, remodeling, and findings of intravascular thrombosis. The cumulative response of these pathological events results in increased vascular resistance within the pulmonary vasculature, right ventricular compensation, and, ultimately, progression to right ventricular failure. The neurohumoral axis has been well described for pathological pulmonary vasoconstriction. The endothelin and renin-angiotensin systems have been shown to mediate pulmonary arterial vasoconstriction. They have been demonstrated to be abnormally active in patients with pulmonary hypertension. The biomarkers of these respective G-protein-based systems can be correlated with the severity and progress of pulmonary hypertension. These *in vivo* findings are the basis for using vasodilators to treat pulmonary hypertension.

**Figure 2:**
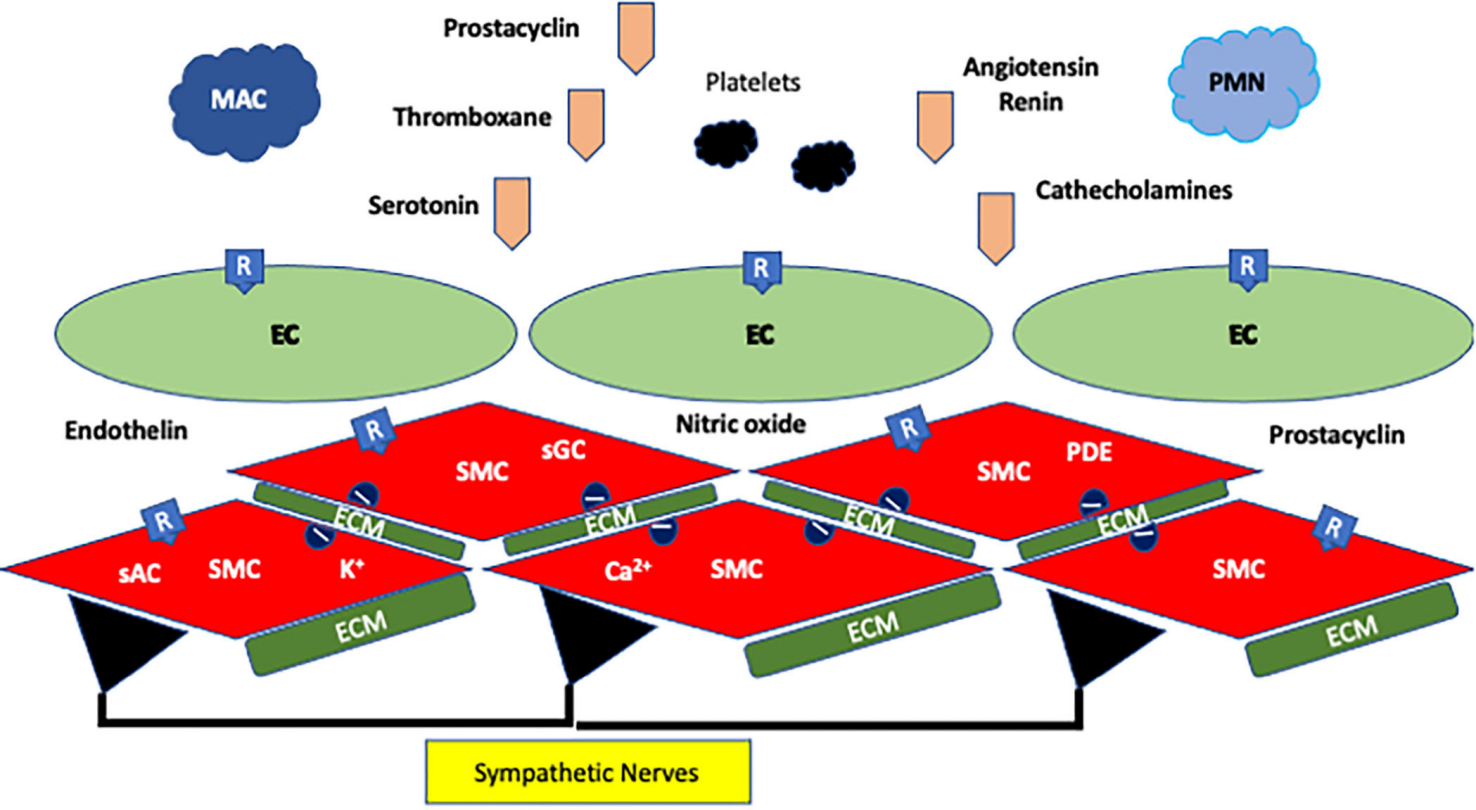
Pathophysiology of pulmonary hypertension. The wall is composed of endothelial cells (EC) and vascular smooth muscle cells (SMC), and these cells sit in an environment of extracellular matrix (ECM). Both types of cells have ion channels for Ca^2+^ and K^+^ and receptors for prostaglandins (thromboxane and prostacyclin) and G-protein agonists (angiotensin, endothelin, serotonin, and catecholamines). In addition, nitric oxide from EC modulates SMCs. Soluble adenyl (sAC) and guanyl cyclases (sGC), and phosphodiesterases (PDE) modulate the ATP and GTP systems. EC and SMC are further influenced by sympathetic nerves in the wall. The combination of activation of EC and SMC, inflammation led by polymorphonucleocytes (PMN) and macrophages (MAC) coupled to progenitor cells, changes in the ECM, surges in or depletion of vasoactive factors and cytokines, altered receptor and ion channel expression, and overactivity of the sympathetic system led to pulmonary hypertension, vessel remodeling, myointimal hyperplasia, and occlusion in the pulmonary vasculature.

In addition to the neurohumoral axis, there is heightened sympathetic nervous system activity in pulmonary hypertension, which has been implicated in the progression of pulmonary hypertension (17). Circulating levels of norepinephrine in plasma have been shown to be directly correlated to pulmonary artery pressure in patients with idiopathic PAH (18). Direct measurement of sympathetic nerve signaling to the muscle circulation by microneurography allows an overall appraisal of a patient's sympathetic drive state. Muscle sympathetic nerve activity (MSNA) is elevated in left heart failure (19, 20). Patients with pulmonary hypertension have also been shown to have increased sympathetic nerve activity in their skeletal muscles compared to healthy controls (21, 22). Velez-Roa et al. demonstrated that MSNA was elevated in patients with PAH and that it was driven by a hypoxic chemoreflex which could be deactivated by inhalation of 100% O_2_ (21). In their estimate, ~25% of the MSNA activity in PAH was driven by the hypoxic chemoreflex. Ciarka et al. examined sympathetic nerve activation over a period of time, and sympathetic nerve activation was found to be associated with deterioration in the patient's clinical condition when assessed by functional and echocardiographic assessments assessed (22). These findings are the basis of pursuing PADN to remove the sympathetic drive and thus reduce ongoing pulmonary hypertension in humans. Figure 2 illustrates the complex pathophysiology of pulmonary hypertension.

## Denervation techniques

Three different catheter-based approaches have been developed, and two have moved into clinical trials: catheter-directed radiofrequency ablation and catheter-directed high-energy ablative ultrasound. In both modalities, the catheter is maneuvered to lie in the main PA. The ablative energy is applied transmurally to the predominantly sympathetic nerve fibers in the adventitia of the PA wall (Figure 1). PADN trials have used predefined anatomical (fluoroscopic) landmarks or been guided by electro-anatomical mapping. More recently, it has been suggested that a more discrete targeting strategy for PADN should be considered where ablation targets areas of increased autonomic nerve activity. It is hoped that such a precision strategy will improve the therapeutic efficacy of PADN and reduce the potential risks associated with empiric ablation of the pulmonary trunk, which may be excessive. Laser ablation remains an experimental catheter-based approach that has not entered human trials.

## Experimental models

The basis of pursuing a sympathetic denervation strategy in pulmonary hypertension has its origins in a body of experimental translation work (Table 2). In 1980, Juratsch et al. (11) demonstrated that after surgical denervation around the pulmonary artery bifurcation areas, there was no hypertensive response within the pulmonary artery after experimental occlusion. Chen et al. (23) used a pulmonary balloon occlusion model to induce significant acute pulmonary hypertension and demonstrated that this hypertensive response could be completely abolished by ablative denervation treatment at the pulmonary trunk and bifurcation and the left pulmonary artery. Importantly, this study demonstrated that PADN was most effective when performed in or near the main pulmonary artery bifurcation. In 2015, Zhou et al. (15) demonstrated that radiofrequency ablation induces severe neuronal injury but is also associated with identifiable intimal injury. However, 30 days after the intervention, the sites of intimal injury were healed and did not demonstrate any intraluminal clot. In addition, at the 30-day follow-up after PADN, nerve transduction velocity remained reduced, suggesting that the pulmonary sympathetic nerve injury was durable. Following PADN, there was decreased expression of FGF-2 and ET-1, growth factors associated with vascular remodeling. In the same report, Zhou et al. (15) showed that PADN led to improved pulmonary hemodynamics, induced pulmonary arterial remodeling, and improved right ventricular function after PADN. Huang et al. (24) demonstrated numerous sympathetic nerves at the pulmonary artery bifurcation and main pulmonary arteries in both rat and human histological specimens. Transthoracic PADN in a rat monocrotaline-induced pulmonary hypertension model successfully ablated the main sympathetic nerves around the pulmonary artery bifurcation. Hemodynamically, pulmonary arterial hypertensive progression was decreased by Transthoracic Pulmonary Artery Denervation due to inhibition of excessive activation of the pulmonary sympathetic nervous system and attenuation of renin-angiotensin-aldosterone system neurohormone-receptor axes. Zhang et al. (25) demonstrated that there was downregulation of α_1_-adrenergic receptors and upregulation of β_2_- adrenergic receptors in pulmonary arteries after PADN; Rothman and coworkers applied radiofrequency PADN in a swine model of pulmonary hypertension and demonstrated that denervation led to reduced mean pulmonary artery pressure and reduced pulmonary vascular resistance; these pulmonary hemodynamic changes were associated with a corresponding increase in cardiac output. These physiological changes could be correlated with the denervation lesions identified in histological samples of the pulmonary arteries. The authors suggested that, based on these findings, the location of the ablation is critical to long-lasting results with PADN (26). However, in their study, Garcia-Lunar et al. (27) performed a porcine pulmonary vein banding model of pulmonary hypertension and divided 18 pigs equally between surgical-PADN, percutaneous-PADN, and sham procedures. Complete transmural PA lesion was demonstrated using surgical clamps for open PADN, whereas only focal damage to adventitial fibers was observed after percutaneous-PADN. However, unlike other studies, the hemodynamic profile of pulmonary hypertension between the three groups did not significantly differ at baseline or follow-up.

**Table 2:** Experimental studies of PADN.

**References**	**Year**	**Species**	**Model**	**Technique**	**Outcome**			
					**PAP**	**PVR**	**Histological**	**Humoral**
Juratsch et al. (11)	1980	Canine	Balloon inflation in main PA	Surgical and chemical PADN	Benefit	Benefit		
Chen et al. (23)	2013	Canine	Left pulmonary distal basal trunk or interlobar artery occlusion	Radiofrequency PADN	Benefit	Benefit		
Zhou et al. (15)	2015	Canine	Intra-atrial N-dimethylacetamide or DHMCT	Radiofrequency PADN			Benefit	
Rothman et al. (26)	2015	Porcine	TxA 2 challenge pre- and post-PADN	Radiofrequency PADN			Benefit	Benefit
Liu et al. (28)	2016	Canine	IV monocrotaline	PADN				Benefit
Zhang et al. (25)	2018	Rat	supracoronary aortic banding	Surgical and chemical PADN	Benefit		Benefit	
Huang et al. (24)	2019	Rat	IV monocrotaline	Radiofrequency PADN	Benefit	Benefit	Benefit	Benefit
Garcia-Lunar et al. (27)	2019	Porcine	Pulmonary vein banding	Surgical and Radiofrequency PADN	No effect	No effect		
Rothman et al. (16)	2019	Porcine	TxA 2 challenge pre- and post-PADN	PADN	Benefit		Benefit	

PAP, Pulmonary Arterial Pressure; PVR, Pulmonary Vascular Resistance; Histological, Histological examination; Humoral, Circulating Growth Factor and Hormonal levels.

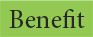
, appropriate response with decrease in the variable.

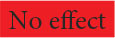
, No appropriate response with decrease in the variable. Gray shade means no data in study for the outcome.

## Human trials

Since 2014, there have been 12 reports on the role of PADN in 490 humans with pulmonary hypertension (311:179; treated: control) (Table 3). Of these, six are case series, three are randomized trials, and three are case reports. Several series are updates of prior studies. Ten studies used percutaneous PADN techniques, and two combined PADN with mitral and/or left atrial surgery. The median follow-up was 12 months (range 2–46 months). The commonest endpoints were 6-minute walking distance and pulmonary artery pressure reduction.

**Table 3:** Human studies.

**Study type**	**References**	**Year**	**Study characteristics**						**Outomes**									
			**Modality **	**n**	**Therapy group**	**Control group **	**Follow up (months)**	**Lost to follow up**	**6-minute walkingdistance**	**Mean pulmonary artery pressure **	**Pulmonary vascular resistance **	**Left ventricular end-systolic diameter **	**Cardiac output**	**Mortality **	**Morbidity **	**Cardiac function**	**Readmission rate **	**Lung transplantation free mortality **
Case Series	Chen et al. (29)	2014	PADN	21	13	8	3	0	1	1								
Case Series	Chen et al. (29)	2015	PADN	66	66	0	12	0	1	1	2	2	2	2		2	2	
Case report	Kiuchi et al. (30)	2015	PADN	1	1	0	6	0	1	2	2	2	2					
Case report	Zhang et al. (31)	2016	PADN	1	1	0	12	0	1	1								
Randomized	Karaskov et al. (32)	2017	Surgery +DN	30	30	15	15	0		2	2							
Randomized	Zhang et al. (33)	2019	PADN	98	48	50	6	0	1	1	2	2	2	2		2	2	2
Case Series	Trofimov et al. (34)	2019	Surgery +DN	140	51	89	2	0	2			2						
Randomized	Romanov et al. (35)	2020	PADN	50	25	25	12	0	2	2	2		2					
Case Series	Rothman et al. (36)	2020	PADN	23	23	0	6	0	2	2	2			2	1			
Case report	Goncharova et al. (37)	2020	PADN	3	3	0	12	0	1	2	2							
Case Series	Witkowski et al. (38)	2020	PADN	10	10	0	6	0	1	2	2							
Case Series	Zhang et al. (39)	2022	PADN	120	120	0	46	0	2									1

PADN, Catheter Pulmonary artery denervation; Surgery and DN, Mitral and/or left atrial surgery with Pulmonary artery denervation.

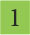
, Well defined primary endpoint.

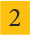
, Secondary endpoint. Gray shade means no data in study for the outcome.

In their First-in-Man trial, Chen and colleagues recruited 21 patients with pulmonary arterial hypertension between March 2012 and May 2012 (23). Thirteen of these patients underwent PADN and were compared to a control group of eight patients who refused the PADN procedure. In this study, PADN was directed at the bifurcation of the main PA and the ostial right and left PA. The study examined three treatment levels (“*level 1:* <*2 mm distal to the orifice of left PA; level 2:* <*2 mm proximal to bifurcation level; and level 3:* <*2 mm distal to ostial right PA”*) of pulmonary artery were selected for intervention (23). At the 90-day follow-up, when compared with the control group, the treated patients demonstrated a marked reduction in mean pulmonary arterial pressure, a significant improvement in their 6-m walking test, and an improved *Tei* echocardiographic index (23). In phase II PADN-1 trial, which ran from April 2012 to April 2014, 66 consecutive patients with pulmonary hypertension who were prospectively enrolled underwent PADN and were followed up for 1 year (29). Hemodynamic success was achieved in 94% of the patients. The patients demonstrated an improved 6-minute walk distance after PADN. In this clinical study, there were eight all-cause deaths, with 75% ascribed to sequelae of pulmonary hypertension, and 12% of patients saw a worsening of their pulmonary hypertension.

In a study by Romano et al. (35), 50 patients with chronic thromboembolic-induced pulmonary hypertension who had undergone a pulmonary endarterectomy were allocated randomly to best medical therapy (*n* = 25) or a PADN intervention (*n* = 25) using remote magnetic navigation. At 1-year follow-up, the patients who received PADN had a notable decrease in their pulmonary vascular resistance, which was accompanied by a demonstrated improvement in the 6-min walk test. In a study by Zhang et al. (33), 98 patients combined with pre- and postcapillary pulmonary hypertension were assigned randomly to receive percutaneous PADN or the best medical therapy with sildenafil coupled with a sham percutaneous PADN. At 6-month follow-up, the authors reported an improvement in the 6-min walk distance and that PADN was associated with a markedly lower pulmonary vascular resistance than in the sildenafil group, which was statistically significant.

The TROPHY1 (Treatment of Pulmonary Hypertension 1) was a multicenter, open-label, early feasibility study that employed an intravascular ultrasound catheter for pulmonary denervation in 23 patients. A maximum of 18 activations were delivered to non-overlapping discrete segments of the main (*n* = 8), right (*n* = 8), and left (*n* = 2) pulmonary arterial walls (36). There were no procedure-related serious adverse events reported. The authors noted a sustained decrease of nearly 20% in pulmonary vascular resistance at 4- or 6-month follow-up. In concert with the hemodynamic improvement, there was an associated increase in the distance achieved on the 6-min walk test. Patients also reported improved daily activity, which was measured by daily step monitoring.

A recent meta-analysis reported PADN results in 339 patients drawn from five controlled trials (40). The reviewers assessed the quality of evidence as Level B. The meta-analysis indicated that compared with the control group, PADN treatment could improve the 6-min walking distance of pulmonary hypertensive patients, could reduce mean pulmonary artery pressure and pulmonary vascular resistance (PVR), and was associated with improved cardiac output. The meta-analysis did not demonstrate a significant effect on the left ventricular end-systolic diameter, patient readmission rate, or patient mortality.

There remain significant limitations to the current set of studies. Patients and responsible physicians were not blinded, the protocol follow-up and outcomes measured were not well regimented, and the potential effect of the placebo could not be quantified. Monotherapy after PADN was prescribed for many patients. Additionally, sample sizes were small; therefore, randomization did not completely balance all baseline characteristics. Maximal medical therapy was not standard in those patients used as controls, and many studies used patients with a spectrum of pulmonary etiologies. Many patients did not complete the expected follow-up. These limitations can be resolved with well-planned, randomized multicenter clinical trials with appropriately treated control groups, enough to allow for clinically meaningful interpretation.

## Current practice

In clinical practice and trials, the current inclusion criteria for PADN are all patients 18 years and older with an mPAP ≥ 25 mm Hg, a PCWP < 15 mm Hg, and a PVR > 3.0 Woods units. General exclusions for PADN are patients with an estimated life expectancy of < 12 months and those who are pregnant and breastfeeding. Specific procedural exclusion criteria include patients diagnosed with WHO pulmonary hypertensive classes II, III, IV, and V (Table 1), severe renal dysfunction with a creatinine clearance of <30 ml/min, and an abnormal platelet count of <100,000/L. The diagnosis of autoimmune diseases, malignancy, tricuspid valve stenosis, and supra-pulmonary valve stenosis are also exclusionary criteria. In general, PADN is recommended to be performed in three areas at the confluence of the main pulmonary trunk and the left main pulmonary artery. At each contact point, the RFA system is programmed to deliver energy >15 W over a temperature range of 45–50°C for 120 s. It is further recommended that the procedure should be paused for 10 s if the patient complains of intolerable chest pain during catheter activation.

## Current guidelines and PADN

The current clinical guidelines for pulmonary hypertension recommend initial general medical care and aggressive risk reduction (41, 42). Early referral to a center of excellence is recommended for accurate disease diagnosis and risk stratification, followed by optimization with one or more vasodilator therapeutic regimens. Thoracic transplantation remains the definitive therapy. Currently, PADN for pulmonary hypertension is considered investigational and not recommended for use outside of structured clinical trials.

## Conclusion

PADN is a novel intervention that has been demonstrated in animal and human studies to be an effective method for treating pulmonary hypertension by limiting sympathetic activity. Based on the current literature, PADN has been demonstrated to be effective in select patients with pulmonary hypertension. The therapy is worthy of clinical promotion, and additional randomized clinical trials against the best medical therapy are required before it can be incorporated into clinical guidelines.

## Author contributions

All authors listed have made a substantial, direct, and intellectual contribution to the work and approved it for publication.

## Conflict of interest

The authors declare that the research was conducted in the absence of any commercial or financial relationships that could be construed as a potential conflict of interest.

## Publisher's note

All claims expressed in this article are solely those of the authors and do not necessarily represent those of their affiliated organizations, or those of the publisher, the editors and the reviewers. Any product that may be evaluated in this article, or claim that may be made by its manufacturer, is not guaranteed or endorsed by the publisher.
